# Quantitative Accuracy of Low-Count SPECT Imaging in Phantom and *In Vivo* Mouse Studies

**DOI:** 10.1155/2011/197381

**Published:** 2011-03-16

**Authors:** Ciara M. Finucane, Iain Murray, Jane K. Sosabowski, Julie M. Foster, Stephen J. Mather

**Affiliations:** Centre for Molecular Oncology and Imaging, Barts Cancer Institute, Queen Mary University of London, London EC1M 6BQ, UK

## Abstract

We investigated the accuracy of a single photon emission computed tomography (SPECT) system in quantifying a wide range of radioactivity concentrations using different scan times in both phantom and animal models. A phantom containing various amounts of In-111 or Tc-99m was imaged until the activity had decayed close to background levels. Scans were acquired for different durations, employing different collimator pinhole sizes. VOI analysis was performed to quantify uptake in the images and the values compared to the true activity. The phantom results were then validated in tumour-bearing mice. The use of an appropriate calibration phantom and disabling of a background subtraction feature meant that absolute errors were within 12% of the true activity. Furthermore, a comparison of *in vivo* imaging and biodistribution studies in mice showed a correlation of 0.99 for activities over the 200 kBq to 5 MBq range. We conclude that the quantitative information provided by the NanoSPECT camera is accurate and allows replacement of dissection studies for assessment of radiotracer biodistribution in mouse models.

## 1. Introduction

*In vivo* imaging of radiopharmaceutical uptake by single photon emission tomography (SPECT) in small animal models is an important tool with great potential for the development of new and improved radiopharmaceutical agents for targeted diagnosis and therapy of cancer [1–3]. In addition to providing high-quality images for qualitative interpretation, accurate quantification is required to determine the amount of uptake in tissues of interest and the pharmacokinetics of a compound *in vivo *[4]. SPECT studies are advantageous as they reduce the number of animals required compared to necropsy studies and allow longitudinal studies in the same animal [5]. 

Radionuclide imaging techniques such as SPECT and positron emission tomography (PET) are widely used for preclinical radiopharmaceutical development [6, 7] because of the possibility of direct translation of results from preclinical laboratories to the human clinical setting [8]. PET imaging has been seen as the more accurately quantifiable technique due to its higher sensitivity; however, development of SPECT imaging systems in recent years has led to higher resolution and sensitivity capabilities, making it an attractive option for quantitative *in vivo* imaging using longer-lived gamma-emitting radionuclides [8].

Three types of radionuclide quantification study are described in the literature. The first is a semiquantitative comparison of different regions of the image such as measurement of target-to-background uptake. The second is quantification of physiologic parameters such as perfusion rate in dynamic studies. The third type, and the focus of this paper, is absolute physical quantification, the measurement of the absolute activity concentration found in a volume-of-interest (VOI) [6, 9]. A number of physical quantities influence the absolute quantification of SPECT images. These include photon attenuation, scatter, and partial volume errors [9]. 

Forrer et al. [10] previously found quantification *in vivo* using the SPECT imaging system to be highly accurate. However, they injected approximately 50 MBq of their compound into each animal with a minimum uptake in the organ of interest of 0.74% injected dose per gram (%ID/g) giving an amount of activity in the target organ of around 1.4 MBq. We were interested in exploring the quantification accuracy further by trying to quantify even lower levels of activity. The administered activity of a radionuclide that must be used for an animal imaging study largely depends on its specific activity, the radiolabelling efficiency, and purity of the compound and can range from approximately 1 to 50 MBq. Uptake in organs of interest typically varies from <1 to 30% [1, 11]. Consequently, there is a need to determine the validity of the measurements from SPECT imaging when working with low injected activities and low uptake in organs of interest. 

Our aim was to determine the accuracy of the NanoSPECT/CT preclinical imaging system for achieving absolute quantification data with both high and low activity concentrations and to use the results in protocol design for future experiments. We investigated the minimum activity concentration and acquisition duration required to collect enough count statistics for accurate quantitative data by performing phantom and *in vivo* studies. This information can in turn determine required animal imaging times and subsequently will influence experimental design.

## 2. Materials and Methods

### 2.1. Imaging Studies

The NanoSPECT/CT preclinical imaging camera (Bioscan, Inc., Washington DC) is a 4-head gamma camera which also incorporates a cone beam CT imaging system. In order to provide high levels of sensitivity and spatial resolution, multi-pinhole collimators are used. For this study, two sets of collimators were tested with pinhole sizes of 1.4 mm and 2 mm. Both collimator sets had a field of view of 30 × 16 mm and consisted of 9 pinholes per head. The sensitivities of the pinhole collimators are approximately >2200 cps/MBq and >3500 cps/MBq with a resolution of ≤1 mm or ≤1.45 mm for the 1.4 mm and 2 mm apertures, respectively. 

The use of multiple pinholes results in multiple overlapping projections, thus making full use of the detector surface available and maximising sensitivity. However, the nature of these projections means that it is not possible to use Filtered Back Projection as a means of tomographic image reconstruction. Hence, an Ordered-Subsets Expectation Maximisation (OSEM) algorithm [12] is used for all tomographic reconstructions. This algorithm does not include any correction for either attenuation or scatter. 

Images are not reconstructed with units of radioactivity concentration per se. A conversion from dimensionless image count values to units of radioactivity concentration in megabecquerel is made by the InVivoScope analysis software (supplied with the camera) incorporating a user defined calibration factor stored within the software. Calibrations must be made for each radionuclide-aperture combination as required. 

The camera was calibrated for In-111 and Tc-99m for both 1.4 mm and 2 mm apertures using a syringe filled with a known concentration of activity previously measured in a dose calibrator (VD 404, Veenstra, the Netherlands). The time of the measurement was also noted. Since dose calibrators are known to be less accurate at low activity levels we measured the calibration syringe with high activity, approximately 50 MBq and a known volume (measured by weight) of In-111 or Tc-99m.

The syringe was imaged acquiring a minimum of 100,000 counts per frame and the scan was reconstructed using the OSEM reconstruction algorithm with a 35% Gaussian filter (this is the manufacturer's description of the FWHM of the Gaussian filter) applied to the 2D projection data to reduce noise, 9 iterations, and a 0.4 mm voxel size. To mimic the attenuation caused by the body of the mouse, the syringe was placed inside a 2.5 cm diameter plexiglass phantom filled with water. 

### 2.2. Phantom Measurements

**Figure 1:**
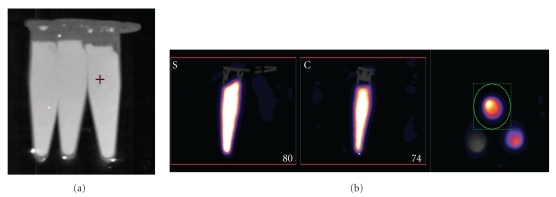
(a) Phantom used in imaging studies. The 3 microcentrifuge tubes were mounted in the centre of a polystyrene holder. The three activity concentrations were approximately 20, 10 (In111)/5 (Tc99m), and 1 MBq/250 *μ*L. (b) Reconstructed image slices with volume-of-interest drawn in the transaxial plane highlighted in green.

A phantom containing a range of activity concentrations was prepared using three 250 *μ*L microcentrifuge tubes. The tubes were completely filled with a total activity of 20 MBq, 10 MBq (In-111) or 5 MBq (Tc-99m), and 1 MBq of either In-111 or Tc-99m in a volume of 250 *μ*L and inserted into a homemade polystyrene support (Figure 1). The radionuclides used for the phantom studies were taken from the same batch of solution used for the calibration. Each tube was weighed before and after addition of the radionuclide to determine the exact volume present. The radioactivity present in each microcentrifuge tube was calculated by weight based on the measured concentration in the calibration syringe.

For the In-111 studies, the phantom was imaged every day for 11 days until the activity present in the low activity microcentrifuge tubes had decayed through approximately 4 half-lives. For the Tc-99m studies, the phantom was imaged at intervals for 2 days (8 half-lives). 

Circular CT scans were acquired in 180 projections, at 45 kVp with 1000 ms exposure time and 3 minute scan duration. The scan range was 26 mm. Helical SPECT scans were acquired with either 30 seconds or 120 seconds per projection frame resulting in acquisition times of either 5 or 20 minutes. Each scan was repeated 4 times to test the reproducibility of the system.

### 2.3. Attenuation Measurements

The attenuation of the phantom versus the syringe used to calibrate the camera was investigated to see how it affected the accuracy of quantifying the image data. This was achieved by measuring weighed amounts (20 MBq) of isotope into either a syringe (1.5 mL) or a microcentrifuge tube (250 *μ*L). The activity present in each was measured using the dose calibrator. The syringe (inside the plexiglass attenuation phantom) and microcentrifuge tube were individually imaged using the same acquisition settings for ten minutes. The resultant files were then reconstructed using the same parameters as the phantom studies and the quantification results were compared. The percentage difference between the two methods was recorded.

### 2.4. Animal Studies (Imaging and Biodistribution)

All animal studies were performed in accordance with British Home Office regulations governing animal experimentation and Advisory Committee for Dangerous Pathogens guidelines in a level 2 containment facility. 5 × 10^6^ A431 cells, transfected with CC-K2 receptors, were injected into the shoulders of 26 female immunodeficient beige SCID mice (Charles River, UK) and allowed to develop into tumours of approximately 5–10 mm in diameter for use in imaging and biodistribution studies. 

10 days after injection, the mice were injected intravenously in the tail with 1 of 12 different In-111-labelled compounds (*n* = 2/3 per condition). Approximately 11–18 MBq in 200 *μ*L was injected into each mouse. Each injected compound was a peptide with different affinities for the CCK-2 receptor expressed by the tumours [13]. The different biodistribution pattern for each compound resulted in a range of uptake values per gram of organ, therefore testing the limits of the quantification accuracy of the camera.

At 4 hours after injection, the mice were anaesthetised with 2% isoflurane gas and 0.5 L/min oxygen and imaged using the NanoSPECT/CT camera. The animals were kept warm throughout the acquisition using a heated bed system (Minerve, France). Whole body SPECT images were acquired in 20 projections over 45 minutes using the 2 mm pinhole collimators, as indicated by the phantom studies. CT images were acquired with a 45 kVp tube voltage in 190 projections over 6 minutes. Images were reconstructed using the same reconstruction parameters as the phantom studies. 

Postimaging the mice were culled by cervical dislocation, and the tumour and kidneys were harvested for quantification analysis. Standards were prepared from a sample of the original injected material and each tissue sample was weighed and counted in a gamma-counter (LKB Compugamma) along with the standards. The percentage injected dose per organ and gram of tissue was determined for each tissue type.

### 2.5. Image Reconstruction and Statistical Analysis

All SPECT scans were reconstructed in a 256 × 256 matrix using HiSPECT (Scivis GmbH, Germany) software. The same reconstruction parameters were applied for all datasets: 35% Gaussian filtering, 9 iterations with a voxel size of 0.4 mm. Image reconstructions were carried out both with and without a “preclean” background correction option provided by the manufacturer. This consists of an algorithm designed to remove isolated single counts from pixels for which the neighbouring pixels contain no counts.

All images were analysed and fused with the CT image using InVivoScope, proprietary Bioscan software. For the phantom studies, a three-dimensional VOI was defined around each tube using the CT image as a reference and the activity present in megabecquerels was calculated. The same VOI was used for all subsequent phantom image analyses. For the animal studies, the uptake in the tumour and kidneys was also quantified using three-dimensional VOIs and the CT as a guide. For tumours with irregular shapes, multiple VOIs were created and the activities were summed together. The percentage injected dose per organ and gram was calculated using the weight of the tissue obtained after dissection. These results were then correlated with the results of the biodistribution study to determine the accuracy of the SPECT camera. All measured activities were corrected for decay and were compared to the true activity. Statistical analysis was calculated using linear regression analysis using the GraphPad PRISM analysis program.

## 3. Results

### 3.1. Phantom Studies

Reconstructed image slices (sagittal, coronal and transversal) of the phantom are shown in Figure 1 with the defined volume-of-interest highlighted in green. The sum of the activity (total activity) in all slices of the tubes was selected for the quantification measurements. The average uptake of four scans for each condition was calculated and used for analysis. We found the system to be reproducible with a coefficient of variation below 1% for activities higher than 0.5 MBq and below 4% for activities lower than 0.5 MBq.

**Figure 2:**
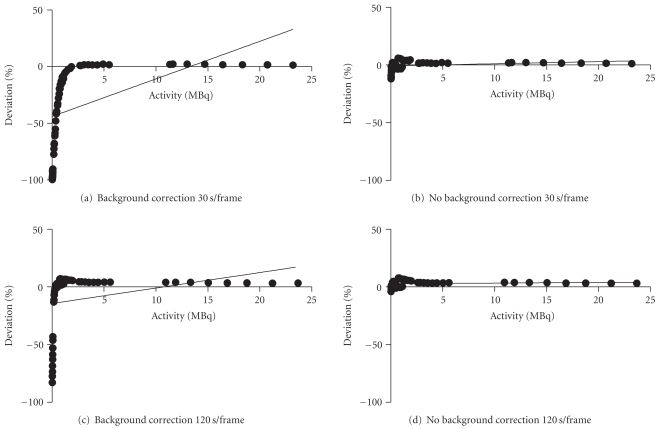
Plot of % deviation of measured activity values from true activity values with Tc-99m using the 1.4 mm pinhole apertures. Each graph represents the full range of activity values measured for all 3 microcentrifuge tubes. The negative bias at low activities introduced by background subtraction is clear in (a) and (c). This bias is eliminated for short and long frame times without any background removal, (b) and (d), respectively.

Initially after reconstructing and quantifying the Tc-99m data with the “preclean background subtraction” included, a significant negative bias was observed at low levels of radioactivity. This was more pronounced for the scans with the shorter frame time. For example, the deviation from true values was within 10% for activities above 0.2 MBq. Below this, the deviation grew significantly to approximately 82% for 0.03 MBq using the longer frame time. However, with the shorter frame time the deviation with 0.2 MBq in the phantom was almost 70% and with activities as low as 0.035 MBq the deviation was 100%. Figures 2(a)–2(d) illustrate the percentage deviation of the measured SPECT data from the true activity in the phantom over a large range of activity using Tc-99m and the 1.4 mm pinhole apertures. It is clear from Figures 2(a) and 2(c) that there is a significant negative bias at low activities with a large deviation from true values (*P* < .001 for 30 s frames and *P* < .01 for 120 s frames). 

The reconstructions were repeated without the default “preclean” subtraction switched on. A marked difference in results was observed. These are shown in Figures 2(b) and 2(d) which represent acquisitions with 30-second or 120-second frame, respectively. With the background correction removed during the reconstruction this negative bias is eliminated and there is no significant deviation from true activity over the whole activity range. The results are also comparable for both the short and long frame times. For both frame times, the observed deviation from the theoretical value for the total activity was consistent over the range of activities imaged. The 120 s frame time activities were within 4% of reference whilst the 30 s frame activities were within 10%. 

The In-111 phantom results for this aperture pinhole size also showed a significant negative bias at low activities with the background subtraction on (*P* < .01). This major negative bias was eliminated after removing the background subtraction; however, the percentage deviation from true activity was still significant even with the background subtraction switched off during reconstruction. As a result, acquisitions and quantification with In-111 using larger pinhole (2 mm) apertures were performed to see if this would improve the results due to the higher sensitivity and ensuing increased count rates of these pinholes.

**Figure 3:**
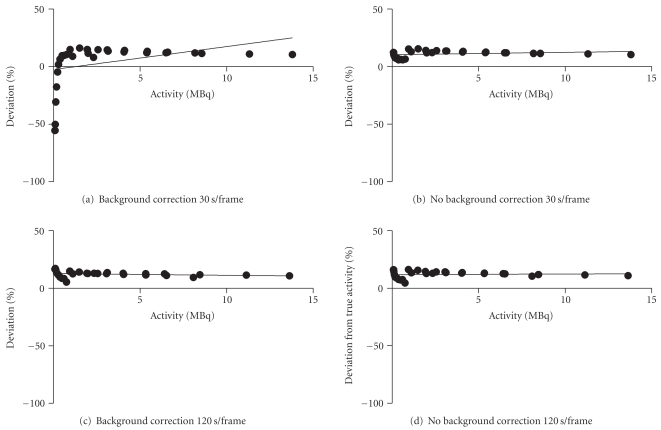
Plot of % deviation of measured activity values from true activity values with In-111 using the 2 mm pinhole apertures. Each graph represents the full range of activity values measured for all 3 microcentrifuge tubes. The negative bias at low activities introduced by background subtraction is also evident for short frame times in (a) whereas in (c) there is no significant deviation due to the longer frame time with a higher number of counts collected. This bias is eliminated for short and long frame times without any background removal, (b) and (d), respectively. An offset of approximately 8–10% exists due to attenuation differences between measurements of the calibration syringe and the samples.

Figures 3(a)–3(d) show the results obtained using In-111 with the 2 mm pinhole apertures. Once more, a significant deviation from true activity was found when the background was subtracted during the reconstruction for the 30 second frame time but interestingly not for the 120-second frame time where even with the background correction switched on there was no significant deviation from true activity over the range of activities. For the scans without background removal there was no significant deviation from true activity values with either the short or the long frame time. For both frame times with the subtraction off, there was a range in deviation of ≤12% across the entire range of activity values, the lowest being approximately 0.087 MBq. It should be noted, however, that the percentage deviation while constant over the range of activities did display an offset of approximately 11%, that is, the deviation was consistently overestimated regardless of scan duration. This is in contrast with the Tc-99m results where the percentage deviation was close to zero. 

### 3.2. Attenuation Measurements

To investigate this offset seen in the In-111 studies, the attenuation of the calibration syringe and the phantom tubes were compared as detailed in the methods section. The difference in absolute quantification of the radioactivity in the phantom tube compared to the calibration syringe was 7.8%.

### 3.3. Animal Studies

**Figure 4:**
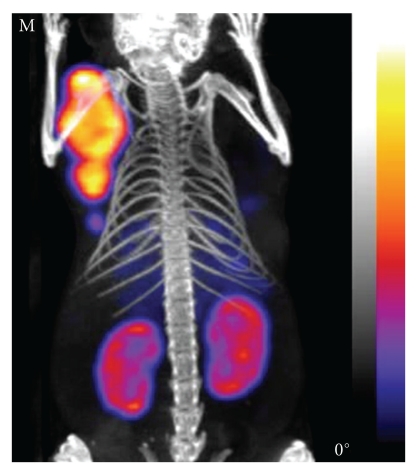
Representative NanoSPECT/CT image of 1 compound showing the tumour and kidney uptake at 4 hours after injection.

**Figure 5:**
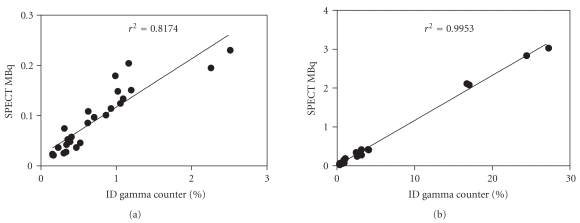
Correlation between SPECT measured results in MBq and *ex vivo* % ID measured in the gamma counter for tumours (a) and kidneys (b). There is a statistically significant correlation between the SPECT data and the biodistribution data which confirms the phantom results that the quantification is reliable at low activities.

All compounds showed uptake in the tumour and kidneys of the injected mice; an example image is shown in Figure 4. The absolute activity present in the organs was calculated from the NanoSPECT/CT data by drawing VOIs using the CT as a reference. After necropsy and counting of tissue samples, the percentage of the injected dose in the tumour and kidney tissues was determined at 4 hours after injection for each mouse (*n* = 26). The uptake values ranged from 0.5–8.4%ID for the tumours and 0.1–93%ID for the kidneys, resulting in very low absolute levels of activity in some of the tumour samples, as little as 0.02 MBq. The results from the necropsy and imaging data sets were then compared. As shown in Figure 5, there is a highly significant correlation between the biodistribution data and the NanoSPECT/CT acquired data for both the tumour (a) and kidneys (b). Linear regression analysis confirmed these observations with *r* ^2^ ≥ 0.8174 for the tumour measurements and *r* ^2^ ≥ 0.9953 and for the kidney measurements.

## 4. Discussion

We have demonstrated that, once the automatic background subtraction feature of the software was disabled, consistent quantification of low activities is feasible with the NanoSPECT/CT preclinical imaging system. It is known that removal of counts from the acquired projection data can lead to non-Poisson statistics [14]. Under these circumstances, the OSEM algorithm which is based upon an assumption of Poisson statistics in the acquired data will become increasingly biased with respect to the quantitation of radioactivity. Furthermore, as the counts acquired are reduced, the background algorithm would seem to be increasingly likely to remove counts originating from the distribution of radiopharmaceutical within the animal/phantom itself.

Although our results demonstrate errors of <12% in absolute quantitation, in particular with regards to In-111, it is important to note that this deviation is consistent over the range of activities imaged. 

In addition, we expanded on our phantom results by performing animal imaging experiments which yielded an excellent correlation with *ex vivo* measurements. The frame times used were as short as 30 seconds resulting in a total scan time of 5 minutes with activities as low as 136 kBq/mL, thereby simulating typical tumour tissue uptake concentrations. This enables us to validate the quantification of animal studies even in situations where the uptake in a target organ is relatively low.

For the In-111 studies, a decrease in pinhole size resulted in increased deviation which was significantly different from the true values. A reason for this discrepancy with In-111 could be because the count rate for In-111 was not as high as with Tc-99m since, due to the higher energy of this isotope the sensitivity of this detector is greater for Tc-99m than for In-111. This inferior count rate will lead to poorer statistics during the reconstruction process and lead to more errors in quantification calculation. A similar pattern of results was observed as with the Tc-99m data but the quantification deviation was higher with the In-111 phantoms. To address this, the phantom study was repeated using 2 mm pinhole apertures. The results of the In-111 experiment with the 2 mm pinholes showed improved correlation. No significant deviation from true activity over the entire range of activities measured was observed, and no difference in deviation for the different frame times was found, even at very low activities. This is likely to be because the larger pinhole size results in an increased sensitivity and higher count rates meaning that scan times can be shorter that those required for smaller pinholes. The reason why there was no significant deviation between the measured and true values for the longer frame time even with the background subtraction switched on is likely to be because of the acquisition of larger number of counts in the image. 

As can be seen in Figure 3, there was a consistent offset for the In-111 phantom studies for both sets of apertures. The offset was approximately 8–11% from true values and was apparent even at high activities. This offset was therefore constant over all activity ranges and frame durations. The offset was also present with the background subtraction either switched on or disabled during the reconstruction. There could be two reasons for this offset. Firstly, this could be a systematic error introduced during the initial dispensing of activity. This is especially relevant for the 1 MBq tube which had a higher offset, as there can be greater uncertainty when measuring out smaller volumes. In fact, this explanation is unlikely as the volumes dispensed were accurately weighed into the phantom tubes to ensure that the exact volume was present. Alternatively, the offset for the In-111 measurement may be due to attenuation differences between the calibration syringe measurements and those of the microcentrifuge tubes. When the camera was calibrated, the syringe was inserted into a 2.5 cm diameter plexiglass phantom to mimic the attenuation of a mouse. However, the microcentrifuge tubes used for the phantom imaging experiments were imaged without such a device and consequently present only a very thin layer of plastic (~0.5 mm) to attenuate the electrons and photons emitted by the radionuclide. It was thought likely that this difference in attenuation of the two types of plastic containers could account for the consistent overestimation of activity values for the phantom studies. After imaging both types of plastic containers containing exactly the same amount activity, a difference of 7.8% was found between the two indicating that attenuation is likely to be the cause of the offset for the In-111 studies. Of note is the fact that this overestimation was not observed during the animal studies, strengthening the hypothesis that this offset was due to differences in the attenuation by the syringe and plexiglass phantoms and that this calibration phantom is a good device for mimicking the attenuation of a mouse.

**Figure 6:**
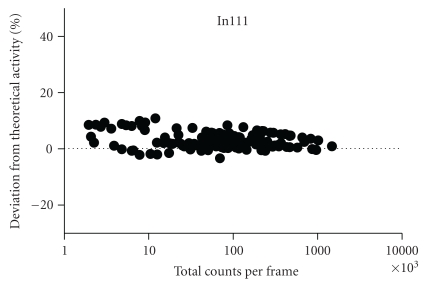
The count rate per second for each activity concentration as a product of frame time (30 s or 120 s) was plotted against the % deviation to determine the optimal total count rate per frame for accurately quantifiable data.

For all the In-111 acquisitions for each pinhole size, the number of counts emitted from each tube in the phantom each day was recorded. This data was normalised for frame time and plotted against the equivalent percentage deviation to determine the optimal total count rate per frame for accurately quantifiable data (see Figure 6).

It can be seen that a minimum of 10,000 counts per frame is required to obtain data that is within 10% of the true activity values when background subtraction is applied. Lower count rates per frame than this will be prone to higher levels of deviation from true activity. With no background subtraction, the percentage deviation was approximately 9.4% with acquisitions of 9,000 total counts per frame and 0.5% with 100,000 total counts per frame when using In-111. Since the count rate, or number of counts per second, is dependent on the activity of the source and the sensitivity of the camera, the frame time can be varied accordingly by the user to achieve a certain total number of counts and, therefore, quantifiable data. This can be used as a guideline for planning future imaging studies in terms of the activity and the scan times required per animal.

Although the results of the phantom studies indicate that the quantification performed with the NanoSPECT/CT scanner is accurate, results obtained from animal studies represent a situation closer to that pertaining to real life. The results from the animal data indicate that there is a highly significant correlation between the measured SPECT data and the *ex vivo* biodistribution data and suggest that results obtained by imaging can potentially replace those obtained from dissection and counting studies. This allows the kinetics and biodistribution of radiotracers to be determined over time in the same animal in longitudinal studies which enables fewer animals to be sacrificed to obtain the same information. The results of the different frame time analyses are also promising because they show that accurate quantification of dynamic imaging of mice using short scan times is achievable. 

One of the limitations of our paper is that it only addresses quantitation of activity in mice using either Tc-99m or In-111. We did not explore the accuracy of measurements in larger animals such as rats or lower energy isotopes such as I-125. In both of these situations, the effect of attenuation will become more significant. For example, in simulations of preclinical imaging without attenuation correction, Chen et al. [15] calculated that imaging of I-125 would result in a deviation of up to 40% from the true activity and Hwang and Hasegawa [16] simulated a 50% reduction in measured activity using I-125 in a rat-sized water phantom which was later confirmed in animal experiments. The emitted low-energy photons (27–35 keV) are more strongly attenuated than with Tc-99m [17]. Wu et al. [18] calculated an underestimation of 10–30% in Tc-99m activity concentration of phantom and rats in studies without attenuation and scatter correction applied. These errors were reduced to −6 to +4% after these corrections were applied. Although we have shown that calibration using an appropriate phantom can correct for the lower levels of attenuation caused by the mouse body when radionuclides of medium-higher energies are used, this may not be the case when either larger animals or lower energy radionuclides are employed. 

## 5. Conclusion

We found that once the automatic background feature of this iterative OSEM algorithm used to implement image reconstruction in this multi-pinhole preclinical SPECT camera was disabled, it resulted in consistent quantitation of Tc-99 m and In-111 over a range of activities and imaging times. The use of an appropriate calibration phantom meant that absolute errors were within 12% of the true activity. Furthermore a comparison of *in vivo* imaging and biodistribution showed a correlation of 0.99 for activities over the 200 kBq to 5 MBq range.

We conclude that the quantitative information provided by the NanoSPECT imager is sufficiently accurate and reproducible to allow replacement of some dissection studies for assessment of radiotracer biodistribution.
